# Quantifying services and disservices provided by insects and vertebrates in cacao agroforestry landscapes

**DOI:** 10.1098/rspb.2022.1309

**Published:** 2022-09-14

**Authors:** Justine Vansynghel, Carolina Ocampo-Ariza, Bea Maas, Emily A. Martin, Evert Thomas, Tara Hanf-Dressler, Nils-Christian Schumacher, Carlos Ulloque-Samatelo, Fredy F. Yovera, Teja Tscharntke, Ingolf Steffan-Dewenter

**Affiliations:** ^1^ Department of Animal Ecology and Tropical Biology, Biocenter, University of Würzburg, Am Hubland, 97074 Würzburg, Germany; ^2^ Alliance of Bioversity International and CIAT, Lima office, Avenida La Molina 1895, La Molina 12, Lima, Peru; ^3^ Agroecology, Department of Crop Sciences, University of Göttingen, Grisebachstr. 6, 37077 Göttingen, Germany; ^4^ Department of Botany and Biodiversity Research, University of Vienna, Rennweg 14, 1030 Vienna, Austria; ^5^ Zoological Biodiversity, Institute of Geobotany, Leibniz University Hannover, Nienburger Straße 17, 30167 Hannover, Germany; ^6^ Universidad Nacional de Piura, Urb. Miraflores s/n, 295 Piura, Peru; ^7^ Universidad Continental Arequipa, Ciencias de la Empresa, Av. Los Incas s/n Urb. Lambramani, José Luis Bustamante y Rivero, Arequipa, Peru; ^8^ Norandino Ltds. Mz X Lote 3 y 4, Zona Industrial II etapa, Piura, Peru

**Keywords:** pollination, vertebrates, arthropods, ecosystem services, shade cover, forest distance

## Abstract

Animals provide services such as pollination and pest control in cacao agroforestry systems, but also disservices. Yet, their combined contributions to crop yield and fruit loss are mostly unclear. In a full-factorial field experiment in northwestern Peru, we excluded flying insects, ants, birds and bats from cacao trees and assessed several productivity indicators. We quantified the contribution of each group to fruit set, fruit loss and marketable yield and evaluated how forest distance and canopy closure affected productivity. Fruit set dropped (from 1.7% to 0.3%) when flying insects were excluded and tripled at intermediate (40%) compared to high (greater than 80%) canopy cover in the non-exclusion treatment. Fruit set also dropped with bird and bat exclusion, potentially due to increased abundances of arthropods preying on pollinators or flower herbivores. Overall, cacao yields more than doubled when birds and bats had access to trees. Ants were generally associated with fruit loss, but also with yield increases in agroforests close to forest. We also evidenced disservices generated by squirrels, leading to significant fruit losses. Our findings show that several functional groups contribute to high cacao yield, while trade-offs between services and disservices need to be integrated in local and landscape-scale sustainable cacao agroforestry management.

## 1. Introduction

Ecosystem services such as pollination and pest control support yields of globally important crops, thus ensuring a considerable part of the world's food supply [1,2]. These nature-based services are biodiversity-driven [3] and provided by multiple animal groups. Vertebrates such as birds and bats, as well as arthropods, may control pest populations [4,5], while bees and many other animals are important crop pollinators [6]. But animals can also cause substantial disservices: some herbivorous arthropod species are pests threatening yields of many crops. Aside from arthropods pests, rodents or other mammals can damage or raid fruits [7,8]. Some animal taxa can be involved in more than one ecosystem service [9], while other taxa are known to provide both services and disservices in the same crop system [10], which can result in management trade-offs. Interactions among services exist as well. For example, beneficial effects of pollination on yields can depend on the level of pest control (e.g. by herbivores lowering attractiveness to pollinators [11]). Therefore, assessing both ecosystem services and disservices is essential to account for potential trade-offs and interactions in biodiversity-friendly and sustainable crop management [12]. Yet, only a handful of studies have addressed multiple services and disservices simultaneously [13,14].

In cacao, a tropical crop grown in agroforestry systems that can be wildlife-friendly [15], multiple animal groups mediate yields. Animal pollination limits productivity: the exclusion of flower visitors can result in fruit set values equal or close to zero [16], even though the identity of pollinator species remains unclear [17]. Pollination gains can be undermined by insect pests causing fruit loss [18], but these pests can be successfully controlled by birds and bats. Yield gains have been attributed to arthropod control by flying vertebrates [19,20]. Other vertebrates, such as squirrels and other rodents, prey on mature cacao fruits and can cause severe harvest losses [8]. Harvest loss can also be due to fungal infections, and by propagating fungal spores, ants can enhance fruit loss [5,10,18]. However, ants can also support yield gains, through reduction in flower and leaf herbivory [5]. Knowledge on combined effects of animal groups is critical to improving our understanding of services and disservices, which in turn might allow developing more efficient management recommendations for profitable and sustainable biodiversity-friendly cacao agroforestry.

The abundance and diversity of services and disservices provided by animals in cacao agroforests are also affected by agroforest and landscape characteristics, such as shade cover and forest distance [5]. Shade cover provided by the canopy of non-cacao trees in agroforests, can improve growing conditions for cacao [21], the prevalence of birds and bats [22], and cacao flower visitation rates [23]. On the other hand, high shade cover can promote the occurrence of pest species and counteract natural pest control [18]. Forest proximity can also influence pest control and pollination, as forest remnants in the landscape provide habitat to many animals, including flying vertebrates and arthropods [24], potential natural enemies of cacao pests. For example, typically, more birds and bats can be found foraging in cacao agroforests closer to the forest than at further distances [22]. As for arthropods, there is evidence of certain cacao flower visitors [25] and ant species [26] being impacted by increasing distance to forest, though this is not consistent across studies [23]. Forest distance and shade cover thus have important implications for biodiversity and the ecosystem functions they provide.

Understanding the complex interactions between animals, the services and disservices they provide, and their dependence on local and landscape characteristics is crucial for aiding decision-making in sustainable cacao agroforestry management. We quantified multiple ecosystem services and disservices in cacao agroforests established in a Peruvian tropical dry forest environment, using exclusion cages and barriers to prevent access of certain animal groups to cacao trees. We excluded flying insects, ants, birds and bats and measured four productivity parameters: fruit set, marketable yield and fruit loss. We analysed fruit loss due to squirrels separately from other fruit loss causes, as these rodents are an important pest species in the study region. Additionally, we assessed how forest distance and canopy cover affected productivity to identify key animal-driven services and disservices.

## 2. Methods

### (a) Study area

We performed the study in 12 organic cacao agroforests located around the farmer community of La Quemazón, in the Piura region of northwestern Peru (5.31° S, 79.72° W, 240 m.a.s.l.; electronic supplementary material, figure S1). The region is characterized by a hot and semi-arid climate, with mean annual rainfall of 235 mm, mostly concentrated between December and March, and a native vegetation cover of submontane, seasonally dry tropical forests [22,27]. To compensate for low water availability in the dry season, agroforests are irrigated by means of gravity-fed flood canals every four weeks on average, depending on water availability

The cacao agroforests ranged in size between 0.3 and 1.1 ha, had comparable cacao planting densities (3 × 3 m or 3.5 × 3.5 m planting grids) and age (5- to 10-year-old) but differed in shade cover (39–84%) and distance to forest (0.1–1.2 km). Shade cover was assessed using a Forest Suppliers spherical densiometer with convex mirror, by averaging the readings of canopy closure (%) in 20 points spread over an area of about 0.15 ha, to obtain a mean value per agroforest. Shade trees were mainly fruit trees such as *Inga* spp., avocado (*Persea americana*), mango (*Mangifera indica*) and mamey (*Mammea americana*) [28].

Distance from each agroforest to the nearest forest (kilometre) was calculated with ArcMap 10.5.1, using a land-use map of Piura [29] updated through ground-truthing [22,28]. The vegetation in the tropical dry forests near the agroforests was scarce in comparison with wet tropical forests. Vegetation was dominated by trees with low diameter at breast height [30], the most common species being *Prosopis* sp. and *Ceiba trichistandra* ([22] and references therein).

### (b) Exclusion experiments

**Figure 1.**
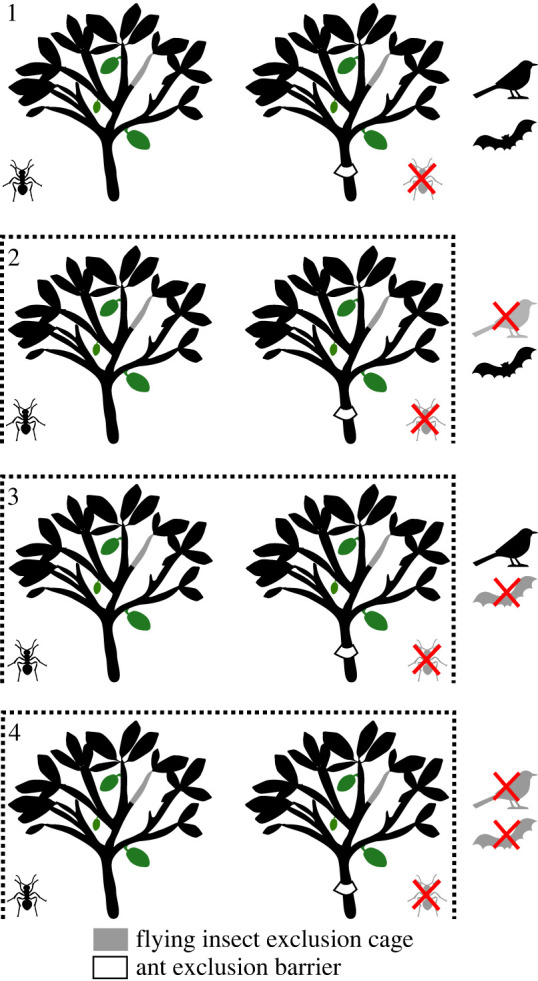
Set-up of experiments in each of the 12 cacao agroforests. Each vertebrate exclusion treatment (1—control, full bird and bat access; 2—closed during the day, no bird access; 3—closed during the night, no bat access; 4—permanently closed, no bird nor bat access) included two cacao trees, one of which was subject to exclusion barriers covered with insect sticky glue to prevent ants' access (ant exclusion). On all experimental trees, flying insect exclusion cages were installed to prevent access of flying insects. To permit ants' entrance, twigs were inserted, but only in trees without ant exclusion. (Online version in colour.)

We established three vertebrate exclusion treatments in September 2019 (figure 1) with exclusion of birds and bats, only birds or only bats, and one open control treatment in each of the 12 selected cacao agroforests and maintained them functional for approximately 1 year, until October/November 2020. Vertebrate exclusions consisted of cages with a size of 2 m wide, 5 m long and 3 m high, each containing two adult cacao trees. Pairs of experimental trees were spaced by 6–9 m, in an area of approximately 0.15 ha. The scaffolds of the structure were made of bamboo poles, and fishing mesh with 2.5 cm openings was used to cover all sides and roof of the cage, preventing the access of birds and/or bats. Selectivity was ensured by differential opening times of each treatment: (1) control treatments consisted of two cacao trees per agroforest left permanently accessible to vertebrates and without a cage constructed around them; (2) bird exclusion cages were kept closed during the day (6.00–18.00) and open during the night (18.00–6.00), to allow the access of nocturnal vertebrates; (3) bat exclusion cages were kept open during the day and closed during the night; (4) full exclusions were permanently closed. Cages were opened and closed manually, every day, for the entire duration of the experiment. By excluding flying vertebrates, we also excluded squirrels (white-naped squirrel; *Sciurus nebouxii*), notorious diurnal fruit predators in the region [31–33]. All trees, including uncaged control trees, were regularly pruned to ensure a standardized tree size throughout the experiment. It is likely that due to the regular pruning, yields on experimental trees were lower than on other trees.

One of the two trees per vertebrate exclusion treatment was subject to an ant exclusion treatment consisting of a vinyl cone located at the base of the trunk, covered with Schacht insect sticky glue, to prevent ants from crawling up the plant from the ground (figure 1). The vinyl cones were tied with rubber tires to the cacao bark at around 30 cm height and isolation foam was stuffed between the cone and the bark (electronic supplementary material, figure S2D). Further, we used cotton wool to stuff cracks, to avoid the smallest ants from crawling up the cacao trees. To also eliminate tree-nesting ants, we applied small doses of a plant-based insecticide Atoxin 15 EC (10 ml l^−1^) with a pipette inside existing ant nests, and when necessary, the application was repeated every two weeks for the entire duration of the experiment. Glue layers were refreshed every two weeks, to prevent the glue from drying out. Experimental trees were pruned regularly, so that the crowns and branches of trees within each cage did not touch each other or the nets, to avoid ant recolonization.

We excluded flying insects from flowers on each of the 96 experimental trees by covering a 35 cm long branch section with UV-stabilized polypropylene gauze (0.5 mm mesh size), supported by an aluminium framework, and sealed with plant wire (figure 1; electronic supplementary material, figure S2B). To permit the access of ants, we inserted little twigs between the nets and the cacao branches, but only in the trees without ant exclusions (electronic supplementary material, figure S2C). Although we aimed to selectively exclude ants only, other crawling insects, such as beetles or bugs could have also entered the exclusion cages through the twig, and likewise, could have been excluded by the ant-barriers.

### (c) Productivity indicators

Every two weeks from November 2019 until October/November 2020, we conducted counts of all recently fertilized fruits (measuring between 1 and 3 cm) and open flowers on each tree. Flower counts started two weeks earlier than the fruit counts and both counts were repeated every 14 days over a period of 1 year. As in other studies [25], small fruits between 1 cm and 3 cm were summed per tree, over the year. Fruits less than 7 days old are smaller than 1 cm and thus not large enough to be reliably monitored. Therefore, we considered only the first 7 days of flowering relevant for calculating fruit set rates. We multiplied the sum of daily flower counts by 7 to obtain an estimate of the total number of flowers that could have given rise to the observed fruits, assuming that flower counts on day one of each 14-day period were representative for the first 7 days. Subsequently, the estimates of small fruits were divided by the total number of flowers, to obtain an estimate of yearly fruit set (%) per tree. Because the decrease in fruit set on the exclusion branches could have been compensated by increasing fruit set on other flowers, outside of the exclusion treatments, as found in other crops [34], the fruit set rates on the tree level that we present here could be slightly overestimated.

Additionally, harvested and lost fruits were counted every two weeks. Squirrel-related fruit loss (%) per tree was established as the proportion of non-harvested mature fruits, i.e. fruits that were large and almost harvestable but were not marketable due to seed predation by squirrels (electronic supplementary material, figure S5). We pooled all other, non-squirrel-related causes of fruit loss (electronic supplementary material, figure S3), i.e. insect damage, germinated seeds or malformed seeds to calculate non-squirrel fruit loss (%). Cacao beans from harvested fruits were dried in the sun and then weighed with a 0.01 g pocket scale to obtain a final measurement of dry weight. The dry weight per tree (kilogram) was summed per tree over all counts (over a period of 1 year) and then multiplied by the number of trees/ha typical for our study area (1100 cacao trees, at a 3 × 3 m planting grid) to obtain a total yield value (kg ha^−1^).

### (d) Data analysis

We constructed generalized linear mixed effect models (GLMM) using R Statistical Software [35] in R Studio 4.1.2 [36] to evaluate the effects of our exclusion treatments on productivity indicators. All models were assembled in the ‘glmmTMB' package [37]. Diagnostic plots and tests for overdispersion and zero-inflation were done with the ‘DHARMa' package [38], adapting the probability distribution when necessary. Model performance indicators were extracted with package ‘performance' [39] and Wald *χ*^2^-tests (Anova type II) reported were conducted with package ‘car' [40]. Predictions were obtained with package ‘ggeffects' [41].

We used a traditional null hypothesis testing approach in which we only included ecologically relevant fixed effect variables and interactions. We restricted ourselves to *a priori* hypotheses and two-way interactions to avoid overparameterizing our models. In all models, shade cover and forest distance were scaled, i.e. the values were subtracted by the mean and divided by standard deviation. In the first model, we assessed the effect of exclusion treatments and farm characteristics (shade cover and forest distance) on cacao fruit set rates. We used a betabinomial distribution with logit link function, using flowers as weights and site as random effect variable. Flying insects, ants and vertebrate exclusions, as well as canopy closure and forest distance were included as fixed effects. We also included two-way interactions of flying insect exclusion with canopy closure, forest distance, ant exclusion and vertebrate exclusion, as each of these parameters could affect the way insect exclusion altered fruit set. E.g. canopy closure and forest distance can affect insect abundances directly, and since ants and vertebrates might be involved in predator–prey relations with flying insects, we considered those the interactions of interest for the fruit set model.

Second, we evaluated changes in cacao fruit loss due to squirrels (squirrel fruit loss/mature fruits), using a model with binomial distribution and logit link, using numbers of mature fruits per tree as weights. Fixed effect variables included were ant exclusion, vertebrate exclusion, canopy closure and forest distance, as well as the two-way interactions between the exclusion treatments and forest distance and canopy closure, respectively. We considered the interaction of ant and vertebrate exclusion not meaningful, because other, non-squirrel related fruit loss cannot be detected when pods are attacked by squirrels. Therefore, this interaction was left out of the analysis. Third, cacao fruit loss due to other causes (non-squirrel fruit loss) was analysed with a similar model as for squirrel-related fruit loss, the only difference being the inclusion of the vertebrate and ant exclusion interaction in this model. We assumed the interaction could be meaningful, for example when birds and bats have different ant predation rates. Fourth, we modelled cacao yield with a hurdle-gamma model (ziGamma), a distribution used to model continuous data with non-constant error that allows zero as a response, overcoming the restriction of a classical gamma distribution to strictly positive observations [42]. We included site as random effect variable; all other fixed effect variables and their interactions were included as in the non-squirrel fruit loss model.

## 3. Results

**Figure 2.**
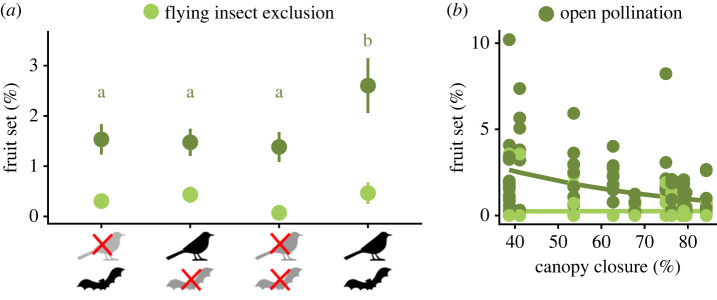
Yearly fruit set rates per tree (mean ± s.e., dots and whiskers) as a function of (*a*) flying insect and vertebrate exclusion, and (*b*) flying insect exclusion and canopy closure. Flying insects were excluded from flowers on the branch level, whereas vertebrates were excluded from trees. Fruit set rates under flying insect exclusion (light green) were measured at the branch level; fruit set of open controls was measured at the tree level (dark green). Letters refer to differences between vertebrate exclusion cages in openly pollinated flowers (emmeans; *p* < 0.05). No differences between vertebrate exclusion treatments were found when flying insects were excluded from branches. For statistics, table 1. (Online version in colour.)

In total, 3337 young cacao fruits developed in total (mean per tree: 35.5 ± 3.0). Only 702 fruits fully matured, 596 of which were harvested, 52 were lost due to squirrel seed predation and 54 were lost due to other, non-squirrel related causes. Average yield was 220.0 ± 23.9 kg ha^−1^ (electronic supplementary material, table S1). Mean fruit set rates were 1.7 ± 0.2% for open pollination and 0.3 ± 0.1% for the flying insect exclusion treatment. Mean open fruit set rates doubled from 1.3 ± 0.3% under full vertebrate exclusion to 2.6 ± 0.5% when both birds and bats had access to the cacao trees, irrespective of ant exclusion (figure 2*a* and table 1). In open controls, predicted fruit set decreased with increasing canopy closure, from 3% under intermediate (39%) canopy closure to 1% under high canopy closure (84%, figure 2*b* and table 1).

**Table 1.** Results of type II analysis of variance with generalized linear mixed effects models relating model parameters to fruit set (%), fruit loss (%) and yield (kg ha^−1^). Parameters include exclusion treatments (excl.) of flying insects, vertebrates and ants, and site characteristics (canopy closure and forest distance, both scaled) and two-way interactions. Flying insects were excluded from flowers on the branch level and vertebrates and ants excluded on the tree level. In all models, site is included as random factor. d.f., degrees of freedom; excl., exclusion. Significance codes: *** *p* < 0.001, ** *p* < 0.01, * *p* < 0.05,° *p* < 0.1.

model parameters	*χ*^2^	d.f.	*p-*value
**fruit set (%)**			
flying insect excl.	51.472	1	**<0**.001***
vert excl.	22.126	3	**<0**.001***
ant excl.	0.854	1	0.355
canopy closure	5.935	1	**0**.015*
forest distance	0.163	1	0.687
flying insect × ant excl.	0.233	1	0.629
flying insect × vert excl.	4.732	3	0.192
flying insect × canopy closure	3.657	1	0.056°
flying insect × forest distance	0.067	1	0.795
**squirrel fruit loss (%)**			
vert excl.	24.265	3	**<0**.001***
ant excl.	1.978	1	0.160
canopy closure	0.531	1	0.466
forest distance	0.319	1	0.572
vert × forest distance	0.365	3	0.947
vert × canopy closure	1.334	3	0.721
ant × forest distance	2.336	1	0.126
ant × canopy closure	0.558	1	0.455
**non-squirrel fruit loss (%)**			
vert excl.	3.573	3	0.311
ant excl.	7.785	1	**0**.005**
canopy closure	0.380	1	0.538
forest distance	0.380	1	0.537
vert × ant excl.	1.752	3	0.626
vert × forest distance	3.225	3	0.358
vert × canopy closure	3.813	3	0.282
ant × forest distance	2.157	1	0.142
ant × canopy closure	0.191	1	0.662
**yield (kg ha^−1^)**			
vert excl.	12.192	3	**0**.007**
ant excl.	0.144	1	0.704
canopy closure	0.407	1	0.524
forest distance	0.002	1	0.962
vert × ant excl.	6.486	3	0.090°
ant × canopy closure	3.086	1	0.079°
ant × forest distance	16.854	1	**<0**.001***
vert × canopy closure	2.493	3	0.477
vert × forest distance	3.470	3	0.325

**Figure 3.**
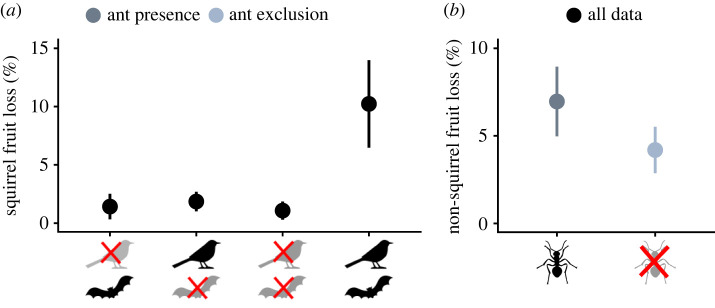
Fruit loss per tree due to squirrels (squirrel fruit loss) as a function of vertebrate exclusion treatments (*a*) and non-squirrel fruit loss as a function of ant exclusion treatments (*b*). Dots and whiskers (means ± s.e., totalled per tree): black, all data; light blue, trees without ants; and dark blue, trees with ants. For statistics, table 1. (Online version in colour.)

**Figure 4.**
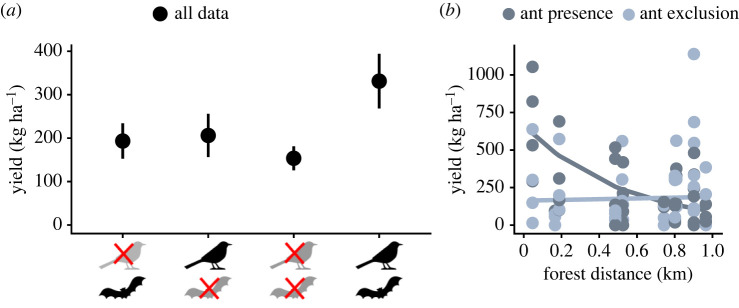
Yield per tree as a function of vertebrate exclusion treatments (*a*) and ant exclusion treatments (*b*). Dots and whiskers (means ± s.e., totalled per tree): black, all data; light blue, trees without ants; and dark blue, trees with ants. For statistics, table 1. (Online version in colour.)

Squirrel fruit loss was highest in the treatments in which all vertebrates, including squirrels, had access to the trees (10.2 ± 3.8%), and was lower when partial and full exclusion treatments prevented squirrel access to cacao trees (figure 3*a* and table 1). Ant access was related to an increase in non-squirrel related fruit loss, from 4.2 ± 1.3% to 6.9 ± 2%, independent of shade cover and forest distance (figure 3*b* and table 1). Yields more than doubled (114% higher) when both birds and bats had access to trees (331.2 ± 62.9 kg ha^−1^, figure 4*a* and table 1), than under full vertebrate exclusion (153.6 ± 27.7 kg ha^−1^). There was weak evidence for an interaction between ant and vertebrate exclusion (table 1). In the presence of birds and bats, yield decreased 28% when ants had access (291.9 ± 79.8 kg ha^−1^), compared to when ants were excluded (374.1 ± 101.0 kg ha^−1^, electronic supplementary material, figure S4). However, in the presence of only birds, ants seemed to benefit yields: their access improved yields by 43%, from 168.2 ± 52.2 kg ha^−1^ to 240.8 ± 83.7 kg ha^−1^ (electronic supplementary material, figure S4). Yield also decreased with distance to forest, but only in the presence of ants, not in their absence (figure 4*b* and table 1). Predicted values ranged from 612 kg ha^−1^ next to the forest to 98 kg ha^−1^ at distances further than 1 km from the forest. No such effect was observed on trees from which ants were excluded (figure 4*b* and table 1).

## 4. Discussion

**Figure 5.**
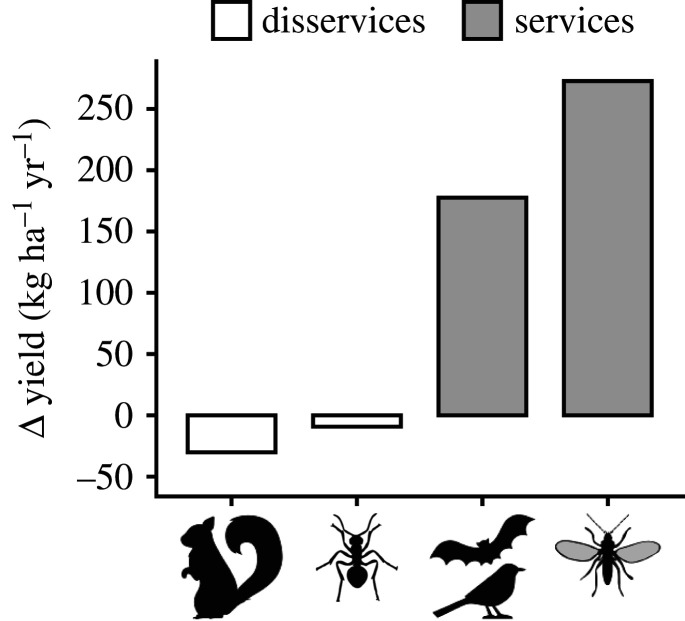
Summary of changes in yield (kg ha^−1^ yr^−1^) associated with the presence of squirrels, ants, birds and bats and flying insects. For clarity and more straightforward calculations, interactions of insect presence with forest distance and canopy closure have been left out here. Ecosystem services (positive yield change) are grey, disservices (negative yield change) white. Due to resource allocation strategies of trees, yield changes due to the presence of flying insects might be overestimated by scaling up findings obtained at branch level to the tree level. For detailed calculations, see electronic supplementary material, methods; for standard errors, see figures 2–4.

Understanding interactions and trade-offs between ecosystem services and disservices of animals is crucial for establishing biodiversity-friendly sustainable management strategies, and to achieve higher-yielding cacao agroforests. Here, we provided a first quantification of the complex interactions between services and disservices in cacao agroforestry. Through our full-factorial experiment, including the year-round assessment of fruit set, fruit loss and yield, we quantified insects and vertebrates' impact on cacao productivity. Fruit set increased when flying insects as well as birds and bats had access to cacao trees and flowers. We also demonstrated a yield increase due to bird and bat access. The effect of ants was twofold: when ants had access, yield increased, but only in agroforests close to forest. Yet, ants also caused minor fruit loss (annually: −9.2 kg ha^−1^). Fruit loss due to squirrels was of bigger importance (annually: −30.1 kg ha^−1^, figure 5). Overall, yield gains due to birds and bats (177.6 kg ha^−1^) and flying insects (272.8 kg ha^−1^) were larger than fruit losses caused by squirrels and ants. Our simultaneous assessment of services and disservices support the design of local and landscape-scale sustainable management strategies that maintain functional biodiversity and maximize benefits for smallholder farming.

### (a) Flying insect services: fruit set increase

Mean fruit set dropped from 1.7% to 0.3% when flying insects were excluded from flowers, underpinning the importance of flying insects as pollinators of cacao that ensure fruit set and yield [16,43]. Therefore, farm management in favour of flying arthropods can likely enhance yield, despite the difficulties associated with scaling up data from the branch to the tree-level and current knowledge gaps about the precise identity of cacao's pollinators [16]. Here, pollination services were better supported by intermediate than high shade cover. This observation is concordant with previous evidence of high yield values in cacao with intermediate shading [5,21] while conserving biodiversity [15]. It is also in line with previous evidence of correlations between shade tree density and abundances of insects that are pollinator candidates, such as ants and Dipteran flies [23]. Considering that abundances of some cacao flower visitors can be promoted by improving habitat conditions [23,44], appropriate shade management might help creating microclimatic conditions that favour flying insect visitors [45], thus enhancing cacao yields.

### (b) Bird and bat services: fruit set increase

Flying vertebrate access enhanced fruit set. However, in the absence of data on arthropod abundances, we can only speculate about the underlying processes. A direct effect through birds and bats pollinating the crop seems unlikely: these vertebrates are much larger than the tiny cacao flowers (1–2 cm intersection). Indirect effects, such as increased pollination and/or reduced herbivory [46,47], are more likely to explain our observations. The large proportion of insectivorous bird and bat species in our study area may control arthropod populations [19,22]. The absence of birds and bats may have resulted in an increased density of mesopredators, which may have reduced the abundance of cacao pollinators. Indeed, exclusion of flying vertebrates has been linked to higher abundances of spiders and ants [20], which in turn may prey on cacao pollinators, causing lower fruit set rates. Further, access of birds and bats to cacao trees is expected to negatively impact the densities of aphids and other herbivores [19], preventing flower damage and potentially fruit abortion, hence increasing fruit set. Similar to other areas, in our study area, sap-sucking arthropods such as aphids and mealybugs are some of the most abundant pests of cacao, as well as phytophagous leaf beetles (Coleoptera: Chrysomelidae) [19,48]. However, their effects on cacao productivity, or the identity of top predators that may control them has not yet been assessed. A lower activity of herbivorous arthropods could result in higher fruit set by increasing the resources that the plant may allocate to fruit production, rather than leaf or flower regeneration [49]. But, detailed data on arthropod densities and food webs is required to test the hypotheses of potential pollination increase and/or herbivory reduction due to the joint access of birds and bats.

### (c) Bird and bat synergistic services: yield increase

Our study showed that birds and bats make a large contribution to cacao yields: their presence increased yield by 114%. The contribution we found, is larger than reported before [5,19], maybe due to the involvement of birds and bats in fruit set rates, and presumably, also in pest control, as in other studies. Both in previous and current studies, the cacao yield increase found in the presence of both birds and bats, was higher than the single benefits provided by birds or bats alone [19]. Such synergistic effects are common when different groups provide complementary ecosystem services [1], as may be the case in this study. It is probable that birds and bats have complementary diets, by consuming insects with different ecological functions. For example, one group could be consuming mostly leaf-consuming insects, while the second one consumes mostly flower herbivores or potential cacao pests [50]. Moreover, the differences in day and night-time activity peaks of the two taxa might allow no enemy-free time for potential cacao pests [51], which might be critical for arthropods whose activity peaks change during their lifetime (e.g. Lepidoptera with palatable larvae) [52]. In order to safeguard and improve birds' and bats’ synergistic contributions to yield, strategies such as creating artificial nesting and roosting spaces for birds and bats could be considered [53,54]. However, benefits of such strategies should be locally assessed because the successes vary across regions [55].

### (d) Ant-related services and disservices

The contribution of ants to cacao fruit production is complex [5,56,57], probably because their contributions depend on species identity and community properties [10,47]. On one hand, we found higher levels of fruit loss related to ant presence, but at the same time, close to forest cacao yield tended to be higher in trees to which ants had access. By forming symbioses with sap-sucking herbivores, and by propagating fungal infections, ants can provide disservices in cacao [5,56]. Detailed mapping of food webs in cacao agroforests would be required to unravel which of these mechanisms was causing ant-related fruit loss. Despite the ant-related fruit loss, yield benefited from ant access in proximity to forest patches. Presumably, some ant species that provide beneficial services to cacao agroforestry systems are dependent on the forest as a refuge or for reproduction, as forest properties can affect tropical ant communities [58]. Detailed information about the composition of ant communities and changes in function of forest distance in our study area would be needed to confirm this pattern. Owing to the association between forest and the persistence of particular ant species [58], maintaining existing forest patches in agricultural landscapes might be beneficial to enhance cacao yield. Known ways in which ants contribute to cacao fruit development are through pest control or aiding pollination by enhancing visitation of small insect visitors of flowers [4,10], but the functional ecology of ants largely depends on the species [59]. Because of the varied functional ecologies of ants, identifying the role of different ant species will be crucial to confirm the positive combined effect of forest maintenance, ant presence and increased yields [58,60].

### (e) Squirrel-related disservices: fruit predation

We quantified an important disservice of vertebrates in cacao: fruit predation by squirrels caused an average loss of 10% of mature fruits from unmanipulated trees, totalling to 30 kg ha^−1^ annual yield loss. The lower squirrel-related fruit loss in the partial vertebrate exclusion than in the control trees which did not have cages built around them, might indicate that exclusion cages deter squirrels, even when the nets are open. An alternative explanation is that by opening of the nets during dusk and dawn, when squirrels are most active, they avoided the caged trees more than the free-standing ones. Fruit predation by squirrels [32] and other rodents [8] have been reported elsewhere as well, and sometimes even more severe. In Ecuador for instance, fruit losses of up to 30% have been related to the same squirrel species, *Sciurus nebouxii* [32]. Farmers believe that in our study area, squirrel populations have surged due to a combination of habitat loss and reduced abundances of native snakes which could be natural squirrel predators. As such, biocontrol by introduction of natural enemies could be a management option to further investigate. The need for research on realistic management alternatives to minimize squirrel disservices in cacao is underlined by the large harvest losses due to squirrels.

### (f) Summary and conclusion

In summary, we quantified the benefits that insects, birds, and bats provide to cacao yield by improving fruit set rates and marketable yield, but we also showed that squirrels and ant species can provide important disservices by enhancing fruit loss (figure 5). Because the yield losses by ants and squirrels represent significant income losses for farmers (9.2 and 30.1 kg ha^−1^ yr^−1^, respectively), management should aim at minimizing these disservices. Nevertheless, the positive yield contributions by biodiversity surmount the yield losses. Yield gains due to flying insects could mount to 272.8 kg ha^−1^ yr^−1^, whereas birds and bats provide benefits of 177.6 kg ha^−1^ yr^−1^. Our results also show variations in contributions of ants and flying insects, due to forest distance and shade cover (not shown in figure 5). Based on our findings, we propose that biodiversity-friendly and sustainable management should: (1) comprise intermediate levels of shade cover of around 40%, to foster populations of flying insects that are indispensable for fruit set success; (2) maintain or restore forest patches at distances of only a few hundred meters to maintain beneficial effects on marketable yields; and (3) implement management strategies that account for interactions among services and disservices.

## Data Availability

Data are available at https://osf.io/5wgc2. The data are also provided in the electronic supplementary material [61].
